# Next-generation imaging in prostate cancer

**DOI:** 10.3389/fruro.2026.1829407

**Published:** 2026-05-29

**Authors:** Ahmed Rabie, Maurizio Buscarini, Osama Zaytoun

**Affiliations:** 1 Urology, Northwell Health, New York, NY, United States; 2 Urology, Mount Sinai, New York, NY, United States

**Keywords:** artificial intelligence, multiparametric MRI, prostate cancer, PSMA PET, micro-ultrasound

## Abstract

**Background:**

Early detection of clinically significant prostate cancer (csPCa) remains a major challenge in urologic oncology. Although prostate-specific antigen (PSA) screening and systematic transrectal ultrasound– guided biopsy has historically been the main diagnostic approaches, these strategies are associated with both overdiagnosis of indolent disease and underdetection of clinically significant tumors. Recent advances in imaging technologies—including multiparametric magnetic resonance imaging (mpMRI), high-resolution micro-ultrasound (MUS), and prostate-specific membrane antigen positron emission tomography (PSMA PET)—have significantly improved the diagnostic pathway for prostate cancer. In parallel, artificial intelligence (AI)–based algorithms have emerged as promising tools for enhancing image interpretation and reducing diagnostic variability.

**Objective:**

This review aims to summarize current evidence regarding the diagnostic performance of micro- ultrasound, mpMRI, and PSMA PET in prostate cancer detection and to explore the potential role of artificial intelligence in integrating these imaging modalities to improve diagnostic accuracy.

**Methods:**

A systematic literature search was performed using PubMed and MEDLINE databases for articles published between January 2013 and December 2024. The following search strategy was employed using combinations of MeSH terms and keywords: (‘prostate cancer’ OR ‘prostate neoplasm’ OR ‘prostatic carcinoma’) AND (‘micro-ultrasound’ OR ‘high-resolution micro-ultrasound’ OR ‘micro-US’ OR ‘PRI-MUS’ OR ‘ExactVu’) OR (‘multiparametric MRI’ OR ‘mpMRI’ OR ‘multi-parametric magnetic resonance imaging’ OR ‘PI-RADS’) OR (‘PSMA PET’ OR ‘prostate-specific membrane antigen’ OR ‘68Ga-PSMA’ OR ‘18F-DCFPyL’ OR ‘PSMA-11’) OR (‘artificial intelligence’ OR ‘machine learning’ OR ‘deep learning’ OR ‘radiomics’ OR ‘neural network’). Only English-language, peer-reviewed original research articles, systematic reviews, meta-analyses, and major consensus guidelines were included. Case reports, editorials, conference abstracts, and non-peer-reviewed publications were excluded. Two authors (A.R. and O.Z.) independently screened titles and abstracts for relevance. Full texts of potentially eligible articles were retrieved and assessed against predefined inclusion criteria: (1) evaluation of mpMRI, micro-ultrasound, or PSMA PET for prostate cancer detection, localization, or staging; (2) reported diagnostic accuracy metrics (sensitivity, specificity, AUC) or biopsy outcomes; (3) sample size ≥20 patients for original studies. Disagreements were resolved by consensus with a third author (M.B.). Reference lists of included articles were hand-searched for additional relevant studies. A narrative synthesis was conducted due to heterogeneity in study designs, populations, and outcome measures.

**Results:**

Multiparametric MRI has become a cornerstone in prostate cancer diagnosis due to its high sensitivity for detecting clinically significant diseases and its role in guiding targeted biopsies. Micro-ultrasound, offering substantially higher spatial resolution than conventional ultrasound, has demonstrated promising diagnostic performance and may represent a cost-effective alternative or complement to in review mpMRI. Meanwhile, PSMA PET imaging has shown high sensitivity for identifying both intraprostatic lesions and metastatic disease. Emerging evidence suggests that combining these imaging modalities may significantly enhance detection rates. Furthermore, artificial intelligence techniques—including machine learning and deep learning algorithms—have shown potential in improving lesion detection, segmentation, and risk stratification in prostate imaging.

**Conclusion:**

The integration of multimodal imaging approaches with artificial intelligence represents a promising investigational strategy that may, following prospective validation, improve prostate cancer detection and characterization. Future prospective studies are needed to validate these technologies and define their optimal role in clinical practice.

## Graphical Abstract

**Figure:**
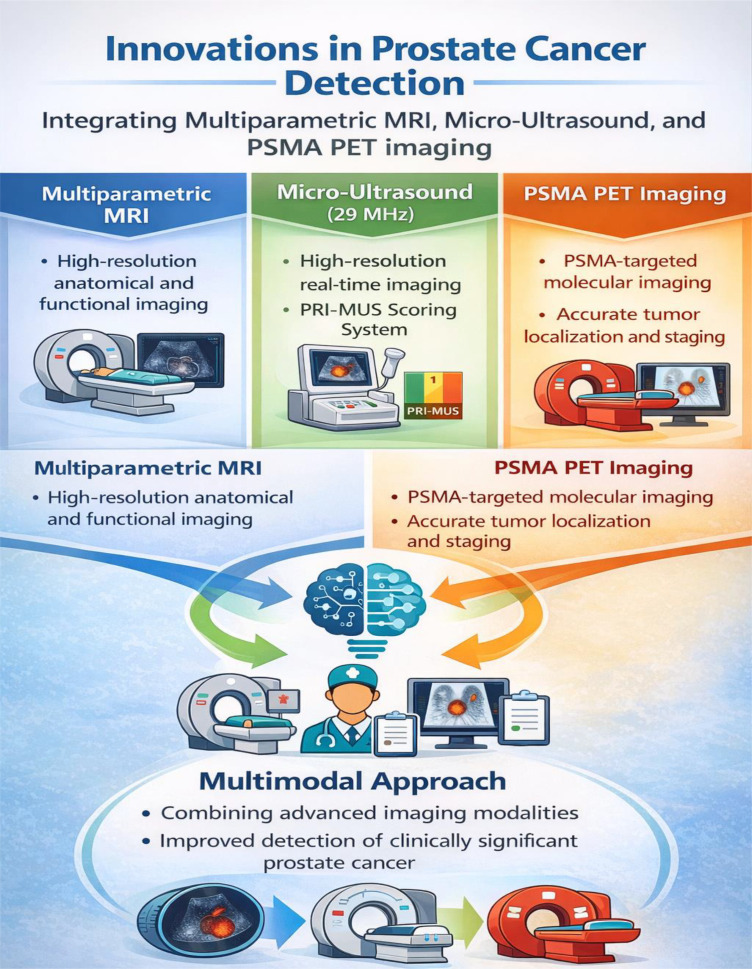

## Introduction

Prostate cancer (PCa) remains one of the most frequently diagnosed malignancies among men worldwide and represents a significant cause of cancer-related morbidity and mortality. According to global cancer statistics, prostate cancer is the second most commonly diagnosed cancer in men and a leading cause of cancer-related deaths worldwide (1). Early detection of clinically significant prostate cancer (csPCa) is critical for improving oncologic outcomes while avoiding overtreatment of indolent disease.

Traditionally, the diagnostic pathway for prostate cancer has relied on serum prostate-specific antigen (PSA) testing, digital rectal examination (DRE), and systematic transrectal ultrasound (TRUS)-guided biopsy. Although PSA-based screening has contributed to a reduction in prostate cancer mortality, this approach has important limitations, including overdiagnosis of clinically insignificant tumors and the potential for missing clinically significant disease during systematic biopsy sampling (2, 3). Consequently, improving the diagnostic accuracy of prostate cancer detection while minimizing unnecessary biopsies remains a major goal in contemporary urologic practice.

Over the past decade, imaging has increasingly assumed a central role in the prostate cancer diagnostic pathway. Multiparametric magnetic resonance imaging (mpMRI), which integrates T2-weighted imaging, diffusion-weighted imaging, and dynamic contrast-enhanced sequences, has significantly improved the detection and localization of clinically significant prostate cancer (4). The development of the Prostate Imaging Reporting and Data System (PI-RADS) has further standardized MRI interpretation and reporting, facilitating widespread adoption of MRI-targeted biopsy techniques (5). Landmark trials such as the PROMIS study demonstrated that mpMRI has superior sensitivity compared with TRUS-guided biopsy for detecting clinically significant prostate cancer, while the PRECISION trial showed that MRI-targeted biopsy improves detection of clinically significant disease and reduces the diagnosis of low-risk tumors (6, 7). As a result, mpMRI is now recommended by major international guidelines as an important step in the diagnostic evaluation of men with suspected prostate cancer.

Despite these advances, mpMRI is not without limitations. Factors such as cost, availability, interobserver variability, and the potential for missed lesions—particularly small tumors or those located in the anterior prostate—remain challenges in clinical practice (8). These limitations have prompted the development of additional imaging modalities that may complement or enhance the diagnostic performance of mpMRI.

High-resolution micro-ultrasound (Micro-US) has recently emerged as a promising imaging modality in prostate cancer detection. Operating at frequencies up to 29 MHz, micro-ultrasound provides substantially higher spatial resolution than conventional ultrasound, enabling improved visualization of suspicious prostate lesions (9). The introduction of the Prostate Risk Identification using Micro-Ultrasound (PRI-MUS) scoring system has facilitated standardized lesion assessment and targeted biopsy guidance. Early clinical studies have suggested that micro-ultrasound may achieve diagnostic performance comparable to mpMRI for detecting clinically significant prostate cancer and may represent a valuable adjunct or alternative imaging modality in the diagnostic pathway (10).

Another rapidly evolving technology in prostate cancer imaging is prostate-specific membrane antigen positron emission tomography (PSMA PET). PSMA is a transmembrane glycoprotein highly overexpressed in prostate cancer cells, particularly in high-grade and metastatic disease (11). Radiotracers such as ^68Ga-PSMA-11 and ^18F-DCFPyL have demonstrated high sensitivity and specificity for detecting prostate cancer lesions. While PSMA PET imaging has become widely established for staging and detection of biochemical recurrence, emerging evidence suggests that it may also play a role in the detection and localization of primary prostate cancer (12). Studies evaluating the combination of PSMA PET with mpMRI have reported improved diagnostic accuracy for identifying clinically significant prostate cancer compared with MRI alone (13).

In parallel with these imaging advances, artificial intelligence (AI) has emerged as a promising tool in medical imaging and cancer diagnostics. Machine learning and deep learning algorithms have shown potential for improving lesion detection, segmentation, and risk stratification in prostate imaging. AI-based tools may help reduce interobserver variability in MRI interpretation and enhance diagnostic performance through automated image analysis (14). Furthermore, radiomics approaches that extract quantitative imaging features from medical images may provide additional insights into tumor characterization and disease prediction (15).

Given the rapid evolution of these imaging technologies, integrating multiple imaging modalities with artificial intelligence may represent a promising strategy to improve prostate cancer detection and characterization. A multimodal imaging approach combining mpMRI, micro-ultrasound, and PSMA PET may provide complementary diagnostic information and enhance the identification of clinically significant disease.

**Figure 1:**
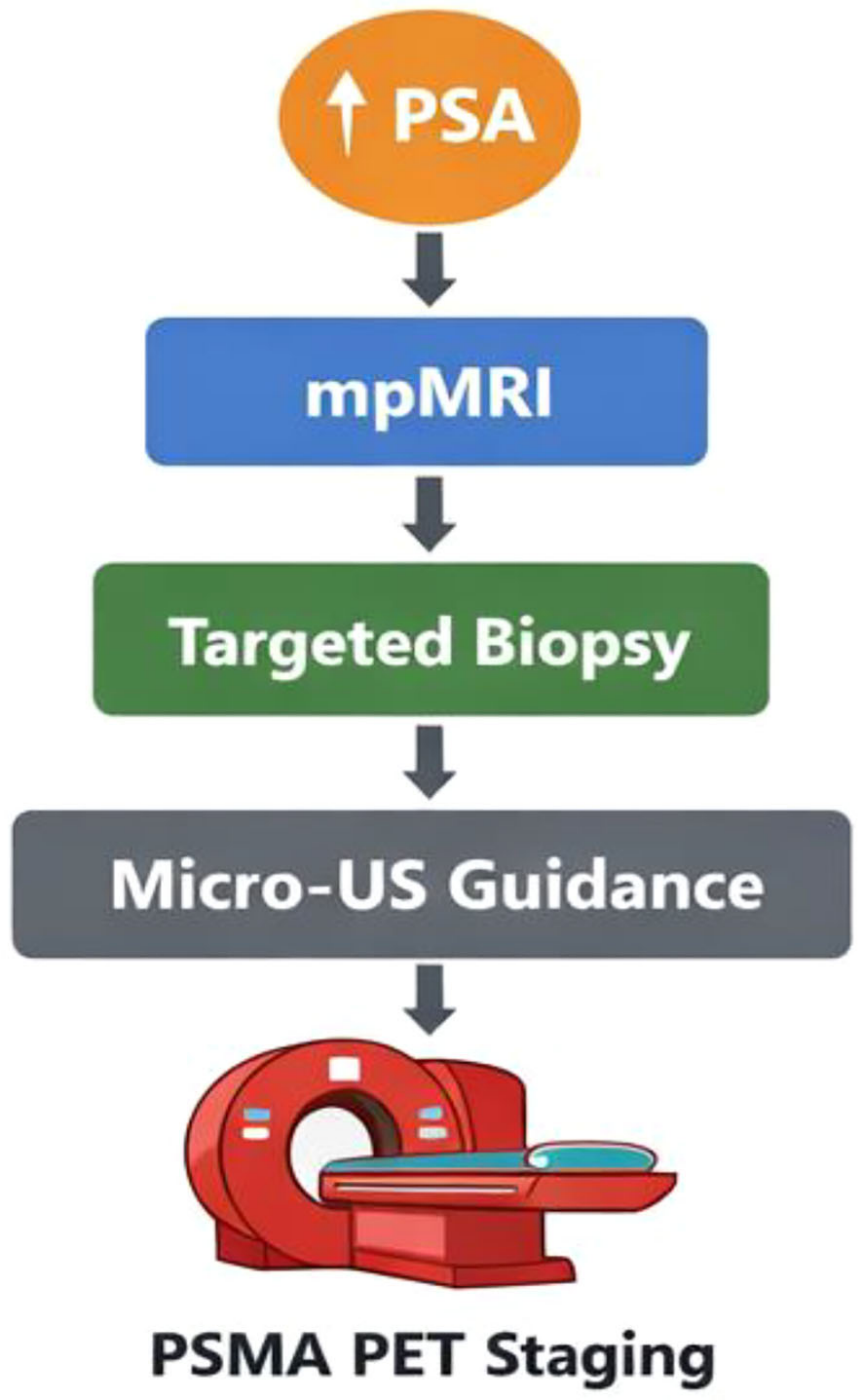
Prostate cancer diagnostic pathway.

Therefore, the aim of this review is to summarize current evidence regarding the role of micro-ultrasound, mpMRI, and PSMA PET in prostate cancer detection and to explore the emerging role of artificial intelligence in integrating these imaging modalities to improve diagnostic accuracy and clinical decision-making as shown in **Figure 1**.

## Multiparametric MRI in prostate cancer detection

Multiparametric magnetic resonance imaging (mpMRI) has emerged as a cornerstone in the diagnostic evaluation of men with suspected prostate cancer. By combining anatomical and functional imaging sequences—including T2-weighted imaging (T2WI), diffusion-weighted imaging (DWI), and dynamic contrast-enhanced imaging (DCE)—mpMRI provides detailed visualization of prostate anatomy and tumor characteristics, enabling improved localization of clinically significant prostate cancer (csPCa) (16).

T2-weighted imaging provides high-resolution anatomical visualization of the prostate and is particularly useful for evaluating lesions in the transition zone. Diffusion-weighted imaging reflects the degree of water molecule movement within tissues and plays a critical role in detecting prostate tumors due to the restricted diffusion typically seen in malignant lesions. Dynamic contrast-enhanced imaging evaluates tumor vascularity and may provide additional diagnostic information, particularly in equivocal cases (17).

To standardize the interpretation and reporting of prostate MRI, the Prostate Imaging Reporting and Data System (PI-RADS) was developed. The current version, PI-RADS v2.1, provides a structured scoring system ranging from 1 to 5, reflecting the probability that a lesion represents clinically significant prostate cancer (5). This standardized framework has improved communication between radiologists and urologists and facilitated the integration of MRI findings into biopsy decision-making.

Several landmark studies have demonstrated the diagnostic value of mpMRI in prostate cancer detection. The PROMIS trial showed that mpMRI has a sensitivity of approximately 93% for detecting clinically significant prostate cancer, significantly higher than the sensitivity of transrectal ultrasound-guided biopsy alone (6). Similarly, the PRECISION trial demonstrated that MRI-targeted biopsy detects significantly more clinically significant cancers compared with standard systematic biopsy while reducing the detection of clinically insignificant disease (7). These findings have contributed to the widespread adoption of MRI-targeted biopsy strategies in modern prostate cancer diagnostics.

MRI-targeted biopsy can be performed using several approaches, including cognitive fusion, MRI-ultrasound fusion biopsy, and in-bore MRI-guided biopsy. MRI-ultrasound fusion biopsy has become particularly popular due to its ability to combine real-time ultrasound imaging with previously acquired MRI data, allowing precise targeting of suspicious lesions identified on mpMRI (18). This targeted approach improves the detection rate of clinically significant tumors while potentially reducing the number of biopsy cores required.

Despite its significant advantages, mpMRI also has certain limitations. Diagnostic performance may be influenced by radiologist experience, image quality, and patient-related factors. Interobserver variability in PI-RADS scoring has been reported, particularly for intermediate-risk lesions such as PI-RADS 3 (19). Additionally, some clinically significant tumors may remain undetected on MRI, particularly small lesions or tumors located in the anterior prostate or transition zone (20).

These limitations have prompted ongoing research into complementary imaging modalities and technological advancements aimed at improving diagnostic accuracy. In this context, high-resolution micro-ultrasound and prostate-specific membrane antigen (PSMA)–based imaging have gained increasing attention as potential adjuncts to mpMRI in prostate cancer detection. Furthermore, emerging artificial intelligence tools may enhance MRI interpretation by improving lesion detection, segmentation, and risk stratification, potentially reducing variability in image interpretation (14).

## Micro-ultrasound in prostate cancer detection

High-resolution micro-ultrasound (Micro-US) has recently emerged as a promising imaging modality for the detection of prostate cancer. Unlike conventional transrectal ultrasound, which typically operates at frequencies of 7–12 MHz, micro-ultrasound utilizes much higher frequencies (up to 29 MHz), providing significantly improved spatial resolution. This enhanced resolution allows better visualization of prostate tissue architecture and potentially facilitates the identification of suspicious lesions associated with clinically significant prostate cancer (csPCa) (21).

One of the major developments enabling the clinical adoption of micro-ultrasound is the Prostate Risk Identification using Micro-Ultrasound (PRI-MUS) scoring system. Similar to the PI-RADS classification used in prostate MRI, the PRI-MUS system categorizes lesions from score 1 to 5 based on their likelihood of representing clinically significant cancer (9). This standardized scoring system has improved the reproducibility and clinical utility of micro-ultrasound-guided prostate biopsy.

Several studies have investigated the diagnostic performance of micro-ultrasound in detecting prostate cancer. Early clinical evaluations demonstrated that micro-ultrasound can identify suspicious lesions with improved clarity compared with conventional ultrasound, potentially allowing more accurate targeting during prostate biopsy (21). In a prospective study evaluating the use of the PRI-MUS protocol, micro-ultrasound demonstrated promising sensitivity for detecting clinically significant prostate cancer and provided improved lesion visualization compared with standard ultrasound imaging (9).

More recently, comparative studies have evaluated the performance of micro-ultrasound relative to multiparametric MRI (mpMRI). Klotz and colleagues conducted a multicenter prospective study comparing micro-ultrasound-guided biopsy with mpMRI-targeted biopsy and reported that micro-ultrasound demonstrated comparable sensitivity for detecting clinically significant prostate cancer (10). These findings suggest that micro-ultrasound may represent a potential alternative imaging modality for lesion detection and biopsy targeting.

In addition to serving as a diagnostic imaging tool, micro-ultrasound offers practical advantages in the clinical setting. Unlike mpMRI, micro-ultrasound imaging can be performed in real time during the biopsy procedure, allowing immediate lesion identification and targeted sampling. This capability may simplify the diagnostic workflow by eliminating the need for MRI-ultrasound fusion platforms and reducing imaging-related costs (22). Furthermore, micro-ultrasound systems are more accessible and may be easier to implement in centers where MRI availability is limited.

Another potential advantage of micro-ultrasound lies in its ability to complement existing imaging pathways. Several studies have suggested that combining micro-ultrasound with mpMRI may improve the detection of clinically significant prostate cancer by identifying lesions that may be missed by one modality alone (23). In particular, micro-ultrasound may be useful for targeting lesions in real time during biopsy procedures following MRI lesion identification.

Despite these promising results, several challenges remain regarding the widespread adoption of micro-ultrasound in prostate cancer diagnostics. The technique is relatively new, and evidence from large multicenter trials is still limited. In addition, operator experience and training may influence diagnostic performance, and further studies are required to determine the optimal role of micro-ultrasound within the prostate cancer diagnostic pathway (24). It is important to distinguish between current evidence and clinical readiness. At present, micro-ultrasound should be considered an emerging technology rather than a standard-of-care alternative to mpMRI. While prospective studies, including the PAIREDCAP and OPTIMUM trials, suggest comparable diagnostic performance, current international guidelines (including EAU and AUA) do not recommend micro-ultrasound as a replacement for mpMRI. The technique remains investigational for primary prostate cancer detection, and further large multicenter trials with standardized protocols are needed before widespread adoption can be endorsed.

Overall, micro-ultrasound represents a rapidly evolving imaging modality that has the potential to improve prostate cancer detection, particularly when integrated with other advanced imaging techniques such as mpMRI and PSMA-based imaging. Future prospective studies are needed to better define its diagnostic performance and clinical role in comparison with established imaging strategies.

## PSMA PET imaging in prostate cancer detection

Prostate-specific membrane antigen (PSMA)–based positron emission tomography (PET) imaging has rapidly emerged as one of the most important advances in prostate cancer imaging over the past decade. PSMA is a type II transmembrane glycoprotein that is highly overexpressed in prostate cancer cells, particularly in high-grade tumors, metastatic disease, and castration-resistant prostate cancer. Because of this high level of expression, PSMA has become an attractive molecular target for diagnostic imaging and therapeutic applications in prostate cancer management (25).

Several radiotracers targeting PSMA have been developed for clinical imaging, with ^68Ga-PSMA-11 and ^18F-DCFPyL being among the most widely used. These tracers allow high-contrast visualization of prostate cancer lesions through PET imaging and have demonstrated excellent diagnostic performance for detecting both primary and metastatic disease (26). Initially, PSMA PET imaging was primarily used for staging high-risk prostate cancer and for detecting biochemical recurrence following definitive therapy. However, growing evidence suggests that PSMA-based imaging may also have a role in the detection and localization of primary prostate tumors.

Recent studies have evaluated the diagnostic accuracy of PSMA PET imaging in patients with suspected prostate cancer prior to biopsy. Early investigations demonstrated that PSMA PET can identify intraprostatic tumor foci with high sensitivity, particularly in patients with high-risk disease (27). Additionally, PSMA PET imaging may help identify clinically significant prostate cancer lesions that may not be clearly visualized using conventional imaging modalities.

One of the most important studies evaluating the role of PSMA PET in primary prostate cancer detection is the PRIMARY trial, a prospective multicenter study that investigated the additive diagnostic value of PSMA PET/CT when combined with multiparametric MRI (mpMRI). The study demonstrated that combining PSMA PET with mpMRI significantly improved the detection of clinically significant prostate cancer compared with MRI alone. Specifically, the combined imaging approach increased sensitivity for detecting clinically significant disease while maintaining acceptable specificity (13).

The integration of PSMA PET with mpMRI has therefore gained increasing attention as a potential multimodal imaging strategy for prostate cancer diagnosis. MRI provides excellent anatomical and functional information regarding the prostate and surrounding tissues, while PSMA PET offers molecular imaging capabilities that allow identification of prostate cancer cells based on PSMA expression (28). When used together, these modalities may provide complementary diagnostic information that improves lesion localization and risk stratification.

In addition to improving primary tumor detection, PSMA PET imaging also offers important advantages for disease staging. Numerous studies have demonstrated that PSMA PET has superior accuracy compared with conventional imaging techniques such as computed tomography (CT) and bone scans for detecting lymph node and distant metastases (29). This improved staging capability may influence treatment planning and guide clinical decision-making in patients with newly diagnosed prostate cancer.

Despite its promising diagnostic performance, several challenges remain regarding the routine use of PSMA PET imaging in the initial diagnosis of prostate cancer. These include cost considerations, limited availability in certain regions, and the need for further prospective trials to define its optimal role within the diagnostic pathway. Additionally, the interpretation of PSMA PET findings requires specialized expertise, and standardized reporting systems are still evolving (30). A clear distinction must be made between the well-established clinical roles of PSMA PET and its investigational applications. PSMA PET is supported by high-level evidence and guideline recommendations for: (1) staging of intermediate and high-risk prostate cancer, (2) detection of biochemical recurrence following radical prostatectomy or radiotherapy, and (3) patient selection for PSMA-targeted radioligand therapy. However, the use of PSMA PET for primary tumor detection in patients with suspected localized prostate cancer—outside of the context of positive mpMRI findings—remains investigation. The PRIMARY trial provides encouraging data regarding the additive value of PSMA PET to mpMRI, but this approach is not currently recommended as a first-line diagnostic strategy in routine clinical practice. Patients should be counseled accordingly when PSMA PET is considered for primary detection, and its use should ideally be restricted to research protocols or specialized centers until further validation is available.

Nevertheless, PSMA PET imaging represents a significant advancement in prostate cancer diagnostics and is increasingly being incorporated into clinical practice. When integrated with other imaging modalities such as mpMRI and micro-ultrasound, PSMA-based imaging may contribute to a more comprehensive and accurate diagnostic pathway for prostate cancer detection.

## Comparative performance of imaging modalities

The key characteristics, strengths, and limitations of mpMRI, micro-ultrasound, and PSMA PET imaging are summarized in **Table 1**.

**Table 1:** Comparison of imaging modalities for prostate cancer detection.

Imaging modality	Principle	Strengths	Limitations	Clinical role
multiparametric MRI (mpMRI)	Anatomical and functional imaging (T2-weighted, diffusion-weighted, dynamic contrast-enhanced)	High sensitivity for clinically significant prostate cancer; standardized PI-RADS reporting system; enables targeted biopsy; no ionizing radiation	High cost; limited availability in some regions; interobserver variability in interpretation; may miss small lesions or tumors in anterior prostate/transition zone	Initial diagnostic evaluation; guidance for targeted biopsy; staging
Micro-Ultrasound (Micro-US)	High-resolution anatomical imaging (operating at frequencies up to 29 MHz)	Real-time imaging during biopsy; lower cost compared to MRI; standardized PRI-MUS scoring system; immediate lesion visualization	Operator dependent; limited long-term outcome data; emerging evidence base; requires specific training	Adjunct to mpMRI; potential alternative when MRI unavailable; real-time biopsy guidance
PSMA PET Imaging	Molecular imaging targeting prostate-specific membrane antigen expression	High sensitivity for metastatic disease and biochemical recurrence; emerging role in primary tumor detection; excellent for staging	High cost; radiation exposure; limited availability; investigational for primary diagnosis outside of research protocols	Staging of intermediate/high-risk disease; detection of biochemical recurrence; patient selection for PSMA-targeted therapy

## Clinical evidence supporting multimodal imaging

Several clinical studies have evaluated the diagnostic performance of micro-ultrasound, mpMRI, and PSMA PET in prostate cancer detection and staging. Key studies are summarized in **Table 2**.

**Table 2:** Key Clinical studies evaluating micro-ultrasound, mpMRI, and PSMA PET in prostate cancer detection.

Study	Year	Study design	Imaging modality	Sample size	Key findings
PROMIS Trial (Ahmed et al.)	2017	Prospective paired validation	mpMRI vs. TRUS biopsy	576 patients	mpMRI demonstrated 93% sensitivity for csPCa, significantly superior to TRUS biopsy
PRECISION Trial (Kasivisvanathan et al.)	2018	Prospective randomized	MRI-targeted vs. standard biopsy	500 patients	MRI-targeted biopsy detected more csPCa and fewer indolent tumors compared to standard biopsy
PAIREDCAP Study (Lughezzani et al.)	2020	Prospective multicenter trial	Micro-US vs mpMRI	248 patients	Micro-US demonstrated comparable detection rates for csPCa compared to mpMRI-targeted biopsy
OPTIMUM Trial	2023	Prospective randomized trial	Micro-US vs mpMRI	~600 patients	Micro-US-guided biopsy showed non-inferior detection of csPCa compared to MRI-guided strategies
PRIMARY Trial (Emmett et al.)	2021	Prospective multicenter	PSMA PET/CT + mpMRI	291 patients	Combining PSMA PET with mpMRI significantly improved sensitivity for csPCa detection compared to MRI alone (97% vs. 83%)
proPSMA Trial (Hofman et al.)	2020	Prospective randomized trial	PSMA PET vs conventional imaging (CT+bone scan)	302 patients	PSMA PET demonstrated superior accuracy for staging high-risk prostate cancer (92% vs. 65%)
Eiber et al.	2016	Prospective	PSMA PET/MRI	53 patients	Simultaneous PSMA PET/MRI improved primary tumor localization compared to MRI alone

## Artificial intelligence in prostate imaging

Artificial intelligence (AI) has rapidly emerged as a transformative technology in medical imaging, with growing applications in prostate cancer diagnosis and management. In prostate imaging, AI techniques—particularly machine learning (ML) and deep learning (DL) algorithms—have shown considerable potential in improving lesion detection, segmentation, and risk stratification. By analyzing large imaging datasets and identifying complex patterns that may not be easily detectable by human observers, AI systems may enhance diagnostic accuracy and reduce variability in image interpretation (31).

One of the most important applications of AI in prostate imaging involves the analysis of multiparametric magnetic resonance imaging (mpMRI). Several studies have demonstrated that deep learning algorithms can assist radiologists in detecting and classifying suspicious prostate lesions on MRI scans. These algorithms are typically trained using large annotated imaging datasets and are capable of automatically identifying regions of interest and predicting the likelihood of clinically significant prostate cancer (32). In some studies, AI-based systems have achieved diagnostic performance comparable to experienced radiologists in detecting clinically significant prostate cancer. It is important for readers to distinguish between AI tools that have received regulatory clearance for clinical use versus those described in proof-of-concept research studies. Currently, several AI-based systems have received FDA clearance or CE marking for prostate MRI analysis, including software for prostate segmentation, lesion detection, and PI-RADS scoring assistance. Examples include Quantib (Quantib BV), Prostate AI (Siemens Healthineers), and Unid-AI (Ubtech). These tools are designed to assist—not replace—radiologists and have demonstrated variable performance in independent validation studies. In contrast, the majority of AI applications described in the research literature remain experimental, having been trained and tested on single-institution datasets without external validation. The following discussion focuses primarily on experimental literature while noting where regulatory-approved tools exist.

AI tools can also assist in prostate segmentation and automated lesion detection, which may improve workflow efficiency in clinical practice. Automated segmentation algorithms can accurately delineate prostate anatomy and tumor boundaries, enabling improved treatment planning and targeted biopsy procedures (33). Additionally, AI-assisted MRI interpretation may help reduce interobserver variability associated with PI-RADS scoring, which remains a recognized challenge in prostate MRI reporting.

Another emerging field within AI-driven prostate imaging is radiomics. Radiomics refers to the extraction and analysis of large numbers of quantitative imaging features from medical images. These features can include shape, texture, intensity, and spatial relationships within tumor regions. Radiomics-based models have shown promise in predicting tumor aggressiveness, Gleason grade group, and risk of disease progression (34). By integrating imaging-derived radiomic features with clinical and genomic data, AI-driven models may contribute to more personalized risk stratification and treatment decision-making in prostate cancer patients.

Beyond MRI, AI techniques are also being explored in other prostate imaging modalities, including micro-ultrasound and PSMA PET imaging. In micro-ultrasound imaging, AI-based algorithms may assist in identifying suspicious lesions and improving the accuracy of targeted biopsy procedures. Similarly, in PSMA PET imaging, machine learning approaches are being developed to improve lesion detection, quantify tracer uptake, and support automated staging of prostate cancer (35).

The integration of AI with multimodal imaging platforms represents an important future direction in prostate cancer diagnostics. Combining information derived from mpMRI, micro-ultrasound, and PSMA PET imaging may enable the development of advanced AI models capable of synthesizing anatomical, functional, and molecular imaging data. Such multimodal AI systems could potentially improve diagnostic accuracy, optimize biopsy targeting, and enhance clinical decision-making in prostate cancer management.

Despite these promising developments, several critical limitations temper the immediate clinical applicability of AI in prostate imaging and warrant careful consideration. First, the majority of published studies lack rigorous external validation across independent, multi-institutional datasets, raising substantial concerns about overfitting and generalizability to diverse patient populations and imaging platforms. Second, training datasets often exhibit significant selection bias, reflecting the prevalence, demographic characteristics, and imaging features of specific patient populations or scanner manufacturers, which may not represent real-world clinical practice. Third, the ‘black box’ nature of many deep learning models limits interpretability and clinician trust, creating barriers to adoption even when technical performance appears adequate. Fourth, variability in ground truth labeling—including interobserver discordance in histopathology grading and reference standard definitions—introduces uncertainty that propagates through AI model training and validation. Fifth, regulatory approval pathways for AI-based diagnostic tools remain in evolution, and currently, no AI tool for primary prostate cancer detection has achieved widespread clinical adoption. Readers should therefore distinguish between validated commercial products (e.g., those with FDA or CE mark approval for specific indications) and experimental algorithms described in proof-of-concept studies. Addressing these limitations will require large-scale, prospectively collected, multi-center datasets with standardized imaging protocols and histopathological correlation, as well as continued refinement of explainable AI methods that provide clinically interpretable outputs (36).

Nevertheless, AI-driven imaging analysis is expected to play an increasingly important role in the future of prostate cancer diagnostics. When combined with advanced imaging modalities such as mpMRI, micro-ultrasound, and PSMA PET, artificial intelligence may contribute to the development of more precise, efficient, and personalized diagnostic pathways for prostate cancer detection.

## Multimodal imaging integration in prostate cancer detection

Recent advances in prostate cancer imaging have highlighted the potential benefits of integrating multiple imaging modalities to improve diagnostic accuracy. While each imaging technique—multiparametric MRI (mpMRI), micro-ultrasound (Micro-US), and PSMA PET—offers unique advantages, combining these modalities may provide complementary information that enhances the detection and characterization of clinically significant prostate cancer (csPCa).

Multiparametric MRI has become the foundation of the modern prostate cancer diagnostic pathway due to its high sensitivity for detecting clinically significant disease and its ability to guide targeted biopsy procedures. However, mpMRI may miss certain lesions, particularly small tumors or those located in the anterior or transition zones of the prostate (37). In this context, micro-ultrasound may serve as a valuable adjunct imaging modality. The high spatial resolution of micro-ultrasound allows real-time visualization of suspicious lesions during biopsy procedures and may help identify lesions that are not clearly visible on MRI (22).

Similarly, PSMA PET imaging provides unique molecular information that complements the anatomical and functional data obtained from MRI. PSMA-based imaging enables visualization of prostate cancer cells based on their molecular expression profile, which may improve lesion detection and localization. Studies evaluating the combination of PSMA PET and mpMRI have demonstrated improved sensitivity for detecting clinically significant prostate cancer compared with MRI alone (13). Several practical standardization challenges must be addressed before multimodal integration can be widely implemented. First, no standardized protocol exists for the optimal sequencing of mpMRI, micro-ultrasound, and PSMA PET—including appropriate intervals between examinations and the potential influence of prior biopsy on imaging findings. Second, concordance between the three established reporting systems (PI-RADS for MRI, PRI-MUS for micro-ultrasound, and the emerging E-PSMA criteria for PSMA PET) remains poorly characterized, and there are no current guidelines for resolving discordant findings across modalities. Third, spatial co-registration of lesions identified on different imaging platforms is technically challenging, particularly when combining PET/MRI with ultrasound-based systems and may introduce localization errors that affect biopsy targeting. Fourth, variability in scanner hardware, acquisition parameters, and reconstruction algorithms across institutions further complicates the development of universal interpretation standards. Future research should prioritize the development of consensus guidelines for multimodal imaging acquisition, interpretation, and reporting, as well as technical solutions for reliable inter-modality image co-registration.

The integration of these imaging modalities could potentially create a comprehensive diagnostic framework, although this remains hypothetical and requires prospective evaluation. In such a multimodal imaging pathway, mpMRI may serve as the initial imaging modality for lesion detection and risk stratification. Micro-ultrasound can then be used during biopsy procedures to provide real-time guidance for targeted sampling of suspicious lesions. PSMA PET imaging may further assist in confirming tumor localization and evaluating potential metastatic disease in selected patients.

Artificial intelligence may play a key role in facilitating this multimodal integration. AI algorithms have the potential to combine imaging data from multiple modalities and identify complex imaging patterns associated with prostate cancer. By integrating anatomical, functional, and molecular imaging data, AI-driven diagnostic models may significantly enhance lesion detection and improve risk stratification (36).

Although multimodal imaging strategies appear promising, several challenges must be addressed before widespread clinical implementation. These include the need for standardized imaging protocols, improved interoperability between imaging systems, and prospective clinical trials evaluating the clinical impact of multimodal diagnostic pathways. Additionally, cost considerations and accessibility of advanced imaging technologies may influence the feasibility of implementing such approaches in routine clinical practice (38).

**Figure 2:**
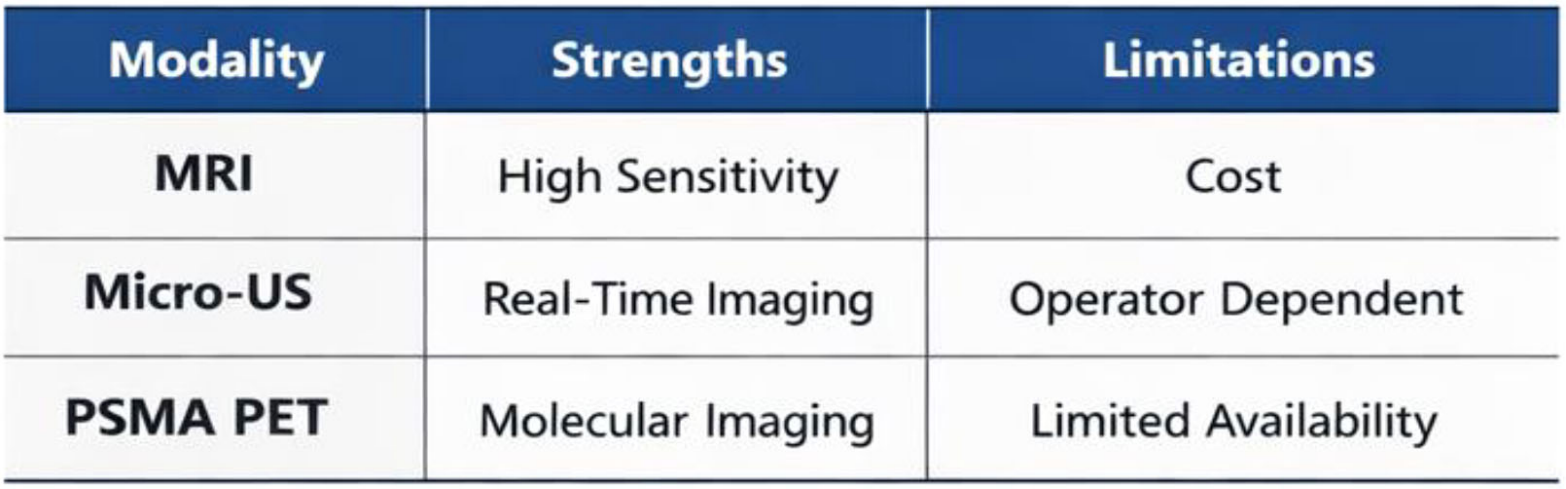
Comparison of imaging modalities.

**Figure 3:**
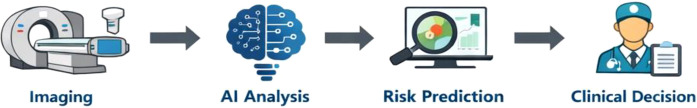
AI-assisted multimodal imaging workflow.

Despite these challenges, the integration of mpMRI, micro-ultrasound, PSMA PET imaging, and artificial intelligence represents a promising direction in the evolution of prostate cancer diagnostics. Future research should focus on validating multimodal imaging strategies and determining their optimal role in clinical decision-making as shown in **Figures 2**, **3**.

## Discussion

Advances in imaging technologies have significantly transformed the diagnostic landscape of prostate cancer over the past decade. While traditional diagnostic approaches relied primarily on PSA testing and systematic transrectal ultrasound–guided biopsy, modern prostate cancer diagnostics increasingly incorporate advanced imaging modalities that enable improved detection and characterization of clinically significant prostate cancer (csPCa). In this context, multiparametric MRI (mpMRI), micro-ultrasound (Micro-US), PSMA PET imaging, and artificial intelligence (AI) represent important developments that may collectively reshape the prostate cancer diagnostic pathway.

Multiparametric MRI has become the cornerstone of contemporary prostate cancer imaging due to its ability to detect clinically significant tumors and guide targeted biopsy procedures. Large prospective studies such as the PROMIS and PRECISION trials demonstrated that mpMRI significantly improves the detection of clinically significant prostate cancer while reducing unnecessary biopsies and the diagnosis of indolent disease (6, 7). As a result, mpMRI is now widely recommended as a key step in the diagnostic evaluation of patients with elevated PSA levels or clinical suspicion of prostate cancer. However, despite its well-established clinical role, mpMRI has certain limitations. Variability in image interpretation, limited availability in some healthcare systems, and the potential for missed lesions—particularly in the anterior prostate or transition zone—highlight the need for complementary diagnostic approaches (19, 20). A methodological limitation that warrants acknowledgment is the considerable heterogeneity among the studies reviewed herein. Patient populations vary substantially across studies, ranging from biopsy-naïve men with elevated PSA to those with known high-risk disease or biochemical recurrence following definitive therapy. Reference standards also differ, with some studies using systematic template biopsy while others employ radical prostatectomy histopathology as the gold standard. Furthermore, definitions of clinically significant prostate cancer (csPCa) are not uniform, with some studies adopting Gleason Grade Group ≥2 and others requiring Grade Group ≥3. This heterogeneity limits direct comparisons of diagnostic accuracy across imaging modalities and underscores the need for prospective head-to-head comparative studies using standardized inclusion criteria and endpoint definitions.

Micro-ultrasound has emerged as a promising alternative or adjunct imaging modality in prostate cancer detection. The higher imaging resolution provided by micro-ultrasound enables improved visualization of prostate tissue architecture and suspicious lesions. Studies evaluating the PRI-MUS scoring system have demonstrated encouraging diagnostic performance for detecting clinically significant prostate cancer (9). Furthermore, prospective studies comparing micro-ultrasound with mpMRI have suggested that micro-ultrasound may achieve comparable sensitivity for clinically significant tumor detection (10). One of the major advantages of micro-ultrasound is its ability to provide real-time imaging during biopsy procedures, potentially simplifying the diagnostic workflow and eliminating the need for MRI-ultrasound fusion systems in some clinical settings.

PSMA PET imaging represents another major advancement in prostate cancer imaging, particularly due to its ability to visualize prostate cancer cells based on molecular expression patterns. Although PSMA PET has traditionally been used for staging and detection of biochemical recurrence, emerging evidence suggests that it may also contribute to primary tumor detection. Studies such as the PRIMARY trial have demonstrated that combining PSMA PET with mpMRI can significantly improve sensitivity for detecting clinically significant prostate cancer compared with MRI alone (13). This complementary relationship between anatomical imaging and molecular imaging highlights the potential value of multimodal imaging strategies.

Artificial intelligence has further expanded the potential of advanced imaging technologies in prostate cancer diagnostics. AI-based algorithms have shown promising results in assisting radiologists with lesion detection, segmentation, and classification on prostate MRI. Deep learning models trained on large imaging datasets may improve diagnostic accuracy and reduce interobserver variability in image interpretation (31, 32). In addition, radiomics-based approaches may enable extraction of quantitative imaging features that provide insights into tumor aggressiveness and biological behavior. The integration of AI with multimodal imaging datasets may therefore represent a powerful tool for improving prostate cancer risk stratification.

Taken together, the integration of mpMRI, micro-ultrasound, PSMA PET imaging, and artificial intelligence may represent the next step in the evolution of prostate cancer diagnostics. Each modality provides unique and complementary information: mpMRI offers high-resolution anatomical and functional imaging, micro-ultrasound enables real-time lesion visualization during biopsy, PSMA PET provides molecular imaging of tumor biology, and AI facilitates advanced image analysis and pattern recognition. Combining these modalities may significantly enhance diagnostic accuracy and improve clinical decision-making. Beyond diagnostic performance, successful clinical implementation of multimodal imaging strategies must address significant practical barriers that currently limit widespread adoption. First, cost considerations are substantial: PSMA PET imaging remains expensive and is not universally reimbursed for primary tumor detection, while mpMRI requires specialized infrastructure and trained radiologists. Micro-ultrasound, while more affordable, has limited availability outside of specialized centers. Second, accessibility varies dramatically across healthcare systems, with advanced imaging modalities concentrated in academic and urban centers, potentially exacerbating healthcare disparities. Third, operator dependence is particularly relevant for micro-ultrasound, where interpretation requires specific training and experience with the PRI-MUS scoring system that is not yet widely disseminated. Fourth, patient burden—including longer examination times, potential need for multiple appointments, and radiation exposure from PET imaging—must be considered in clinical decision-making. Fifth, the cost-effectiveness of multimodal imaging compared with current pathways (e.g., mpMRI with targeted biopsy alone) remains unknown. Until these barriers are systematically addressed through health services research and implementation science, multimodal imaging strategies are likely to remain confined to specialized academic centers and clinical research protocols.

Nevertheless, several challenges remain before multimodal imaging strategies can be widely implemented in routine clinical practice. These include the need for standardized imaging protocols, validation of AI-based diagnostic tools, and prospective clinical trials evaluating the clinical and economic impact of integrated imaging pathways. Additionally, cost considerations and limited availability of advanced imaging technologies may affect accessibility in certain healthcare systems.

Despite these challenges, the rapid evolution of imaging technologies and artificial intelligence continues to expand the possibilities for improving prostate cancer detection and characterization. Future research should focus on validating multimodal diagnostic approaches and determining how these technologies can be optimally integrated into clinical workflows to achieve more accurate and personalized prostate cancer care.

## Future perspectives

The field of prostate cancer imaging continues to evolve rapidly, driven by technological advances in imaging modalities and the growing integration of artificial intelligence into clinical practice. Future diagnostic strategies are likely to rely increasingly on multimodal imaging approaches that combine anatomical, functional, and molecular imaging techniques to improve the detection and characterization of clinically significant prostate cancer.

One promising direction involves the continued integration of multiparametric MRI, micro-ultrasound, and PSMA PET imaging within a unified diagnostic pathway. Advances in hybrid imaging technologies, such as PET/MRI systems, may further enhance the ability to simultaneously obtain high-resolution anatomical information and molecular imaging data, potentially improving lesion localization and staging accuracy (39). In addition, the refinement of micro-ultrasound technology and increased clinical experience may further clarify its role as a cost-effective real-time imaging modality for targeted prostate biopsy.

Artificial intelligence is also expected to play an increasingly important role in the future of prostate cancer diagnostics. AI-driven tools may enable automated lesion detection, improve imaging interpretation, and facilitate risk stratification through the integration of radiomic features with clinical and genomic data. As large multicenter imaging datasets become available, machine learning algorithms will likely become more robust and generalizable across diverse patient populations (31). However, the successful translation of AI tools from research settings to clinical practice will depend on overcoming current limitations related to external validation, algorithmic transparency, and regulatory approval. Prospective clinical trials evaluating AI-assisted versus conventional image interpretation are urgently needed.

Another emerging area of interest involves the development of predictive models that integrate imaging data with clinical variables and molecular biomarkers. Such approaches may enable more precise identification of patients who would benefit from biopsy, active surveillance, or definitive treatment. In addition, AI-based decision support systems may help clinicians optimize diagnostic pathways and personalize prostate cancer management strategies.

Despite these promising developments, further prospective studies are required to validate the clinical utility of multimodal imaging approaches. Large multicenter trials will be essential to determine how these technologies can be integrated effectively into routine clinical practice while maintaining cost-effectiveness and accessibility.

## Conclusion

Advances in imaging technologies have significantly improved the diagnostic evaluation of prostate cancer. Multiparametric MRI has become a central component of the modern diagnostic pathway, enabling improved detection and localization of clinically significant disease. Emerging imaging modalities such as micro-ultrasound and PSMA PET have further expanded the capabilities of prostate cancer imaging by providing high-resolution real-time imaging and molecular characterization of tumors.

Artificial intelligence has also demonstrated considerable potential in enhancing imaging interpretation, reducing diagnostic variability, and enabling advanced image analysis. The integration of AI with multimodal imaging datasets may further improve prostate cancer detection and risk stratification.

A multimodal diagnostic approach that combines mpMRI, micro-ultrasound, PSMA PET imaging, and artificial intelligence may represent a promising but still investigational direction that requires prospective validation in large multicenter trials before clinical implementation can be considered. Continued research and prospective clinical trials will be essential to validate these strategies and determine their optimal role in clinical practice.
